# Supplementary material for: Cellular structure of dinosaur scales reveals retention of reptile-type skin during the evolutionary transition to feathers
Source: Nat Commun. 2024 May 21;15:4063. doi: 10.1038/s41467-024-48400-3 (PMC11109146; doi:10.1038/s41467-024-48400-3)
Supplement: Supplementary file 1 — Supplementary Information [file 41467_2024_48400_MOESM1_ESM.pdf]

SUPPLEMENTARY INFORMATION FOR

Cellular structure of dinosaur scales reveals retention of reptile-type skin during the evolutionary transition to feathers

Zixiao Yang[1,2], Baoyu Jiang[3], Jiaxin Xu[3] & Maria E. McNamara[1,2]

[1]School of Biological, Earth and Environmental Sciences, University College Cork, Cork, Ireland;

[2]Environmental Research Institute, University College Cork, Cork, Ireland;

[3]State Key Laboratory for Mineral Deposits Research, School of Earth Sciences and Engineering and Frontiers Science Center for Critical Earth Material Cycling, Nanjing University, Nanjing, China

Table of contents

Supplementary Notes 1–5

Supplementary Figures 1–13

Supplementary Table 1

Supplementary References

Supplementary Note 1. Taxonomic assignment of specimen NJUES-10

Specimen NJUES-10 preserves in ventral aspect a near-complete and well-articulated skeleton. Features supporting an assignment to the genus *Psittacosaurus* include a short preorbital skull segment (less than 40% of skull length), laterally protruding jugal horns and reduction of the fourth and fifth manual digits[1,2]. To date, four species of *Psittacosaurus* have been reported from the Jehol Biota, including *P. meileyingensis*, *P. lujiatunensis*, *P. major* and *P. mongoliensis*[2]; *P. meileyingensis* and *P. major* were subsequently proposed to be junior synonyms of *P. lujiatunensis*[3]. Most autapomorphies of these species are cranial features[2] that are not visible in ventral aspect. As a result, the assignment of NJUES-10 to existing species remains unclear. Future analyses may shed light on the osteology and species-level taxonomy of the specimen.

Supplementary Note 2. Ontogenetic status of specimen NJUES-10

Accurate age determination of NJUES-10 is not possible due to a lack of histological data. The ontogenetic status of the specimen, however, can be inferred from body proportions. The ontogeny of *Psittacosaurus* is characterised by a shift from quadrupedality to bipedality, with changes in the forelimb-to-hindlimb ratio (the ratio of the combined length of the humerus and radius to that of the femur and tibia) from 0.85–0.84 in hatchlings to 0.71–0.62 in juveniles and 0.66–0.59 in subadults and adults[4].

NJUES-10 has a forelimb-to-hindlimb ratio of 0.67, which falls in the range for juveniles but also closely resembles that of the subadults and adults (Supplementary Table 1). NJUES-10 is considered to represent a juvenile rather than a subadult/adult since the long bone lengths are most comparable to juveniles (Supplementary Table 1). Consistent with this interpretation, NJUES-10 may have been a three-year-old individual based on the recovered relationship between femoral length and age for *P. lujiatunensis*[5].

Supplementary Note 3. Variation in preserved scale morphology in NJUES-10

The preserved skin of NJUES-10 offers an opportunity to compare the gross morphology of the scalation of this specimen with that of other *Psittacosaurus* specimens, in order to provide insights into potential inter-/intra-specific variation. Almost all of the preserved scales are tuberculate (i.e., non-overlapping and non-polarised) and polygonal to rounded basement scales[6] (Supplementary Figs. 2 and 3). Preserved feature scales are rare and occur on the flank of the lower abdomen. The feature scales are rounded and larger than the surrounding basement scales (Supplementary Fig. 3a–b).

In most regions with preserved skin, the basement scales do not form a distinct scale pattern. In the chest region, however, the basement scales show locally an arrangement that includes a central polygonal scale surrounded by smaller, triangular scales (Supplementary Fig. 3c–d). This arrangement is consistent with the hexagram pattern of basement scales common in, and possibly unique to, ceratopsians[7]. Additional notable features occur in the lower abdomen, where quadrangular basement

scales form distinct transverse rows (Supplementary Fig. 3e–f). These scales correspond to the transverse abdominal scales typical of modern crocodilians and some squamates[7].

Prior to this study, most of our understanding of *Psittacosaurus* skin derived from specimen SMF R4970, which shows preserved scaled skin covering almost its entire body[7]. Consistent with NJUES-10, SMF R4970 shows a small number of feature scales in the flank of the thorax and transverse rows of quadrangular scales in the abdomen[7]. The basement scales in SMF R4970 also form a hexagram pattern, but only on the limbs[7] and not in the torso (despite extensive preservation of the basement scales). The presence of the hexagram pattern on the torso of NJUES-10 therefore likely represents inter- or intraspecific variation. The absence of this pattern on the limbs of NJUES-10, on the other hand, may reflect poor skin preservation (Supplementary Fig. 2).

The preserved scales in SMF R4970 are larger than those in NJUES-10. The basement and feature scales are 1.4–2.3 mm and 3–4 mm wide, respectively, in the thorax and abdomen region of SMF R4970, whereas these scales are 0.8–1.2 mm and 1.5–2 mm wide, respectively, in NJUES-10. Given that the femur in SMF R4970 (150 mm; measured from ref. [8]) is almost twice as long as in NJUES-10 (78 mm; Supplementary Table 1), the overall difference in scale sizes likely reflects different growth stages—in modern crocodiles, the scales increase in size with growth[9].

Supplementary Note 4. Skin colour of *Psittacosaurus*

The geometry of the fossil melanosomes in the skin of NJUES-10 (oblate to spheroidal and ca. 0.2–0.4 μm wide) is consistent with previous reports from other *Psittacosaurus* specimens. The latter includes fossilised skin melanosomes in SMF R 4970 (ca. 400 nm long and 250 nm wide)[8], PKUP V1050 (482.5 nm long and 279.3 nm wide)[10] and PKUP V1051 (546.3 nm long and 389.7 nm wide)[10].

The skin melanosomes of *Psittacosaurus* have been interpreted as phaeomelanin-rich based on their ovoid shape; accordingly, the skin of *Psittacosaurus* has been interpreted as a brown colour[8]. A correlation between melanosome geometry and visible tissue colour, however, has been established only for mammalian hair and maniraptoran feathers[10]. This correlation does not hold true for extant reptilian scales[10] and possibly not for the skin of most archosaurs. For instance, in the Nile crocodile[9], the black iguana[11] and the pterosaur *Tupandactylus*[12], melanosomes from the skin morphologically resemble the (low aspect ratio) phaeomelanosomes in extant feathers but are instead rich in eumelanin. Given the lack of chemical evidence for melanin in any specimen of *Psittacosaurus*, it is therefore not possible to determine skin colour.

It is, however, possible to comment on colour patterning. The distribution of fossil melanosomes within the skin varies across the torso (Supplementary Fig. 9). Melanosomes can be absent or can occur in only the uncornified epidermis layer or in both the uncornified and cornified layers (Figs. 4–5 and Supplementary Figs. 6–8). In extant crocodiles, such different distributions correspond to white, intermediate grey and black scale colours, respectively[9]. This lateral variation in melanosome distribution

therefore suggests spatial variation in skin tone. The inferred individual skin tones can persist over 1 mm laterally (Supplementary Fig. 9), indicating at least millimetre-scale, macroscopic colour patterning.

Colour patterning in *Psittacosaurus* has been inferred for specimen SMF R 4970, where the skin is preserved organically[8]. The interpreted pattern is based on observed differences in tone, which were considered to reflect differences in original melanin density[8]. This approach is not applicable to our specimen, in which the skin is mineralised and shows no apparent variation in tone.

It is not possible to characterise in detail the colour pattern in NJUES-10 as this would require extensive sampling of the skin. High-resolution CT with the necessary resolution to visualise the spatial distribution of the mouldic melanosomes is currently not available.

Supplementary Note 5. The lack of feather preservation in NJUES-10

Bristle-like integumentary structures interpreted as feathers have been reported on the tail of specimen SMF R 4970[8,13]. NJUES-10 does not preserve any tail feathers, despite extensive preservation of skin in the torso. In an illustration by Li et al. (Extended Data Figure 3; 2014)[10], two specimens with fossilised skin (PKUPV1050 and PKUP V1051) also appear to lack tail feathers; confirmation of this would require further inspection of the specimens and thus is not considered further here.

The lack of tail feathers in NJUES-10 may be a biological feature, representing sexual, ontogenetic or interspecific variation. Indeed, based on the relationship between femoral length and age[4], NJUES-10 may have been a three-year-old juvenile whereas SMF R 4970 (femur length is ca. 150 mm; measured from ref. [8]) is consistent with a seven-year-old subadult/adult.

Alternatively the apparent lack of tail feathers may be a taphonomic artefact. It has been demonstrated experimentally that, during soft tissue silicification, the volume of silica precipitated correlates with the amount of available silica-binding functional groups, specifically the hydroxyl, amino and carboxyl groups[14]. These functional groups are common in decaying protein-rich tissues that include both scales and feathers. Without decay, however, these functional groups in scales and feathers are likely inaccessible for binding with silica, due to the polymerized and highly cross-linked nature of their protein structure[15]. In NJUES-10, the silicified scales are located almost exclusively in the torso and rarely the limbs and the tail, even though the latter were almost certainly covered by scales *in vivo*[8]. This heterogeneous preservation in the torso suggests heterogeneous rates of decay of the scales between the torso and the limbs/tail. In fact, this pattern is consistent with decay controlled by endogenous microbes[16]. After invading the body cavity from the gut[16], endogenous microbes may have been responsible for degrading the protein structure of the scales in the torso, thereby creating new molecular sites for binding silica[14,15]. Feathers (and scales) from the limbs and tail, on the other hand, are relatively distal to the primary source of decay microbes and may have had experienced limited decay relative to the scalation of the torso. As a result, scales in the limbs and tail would have possessed fewer functional

groups available for binding silica and thus a lower potential for silicification.

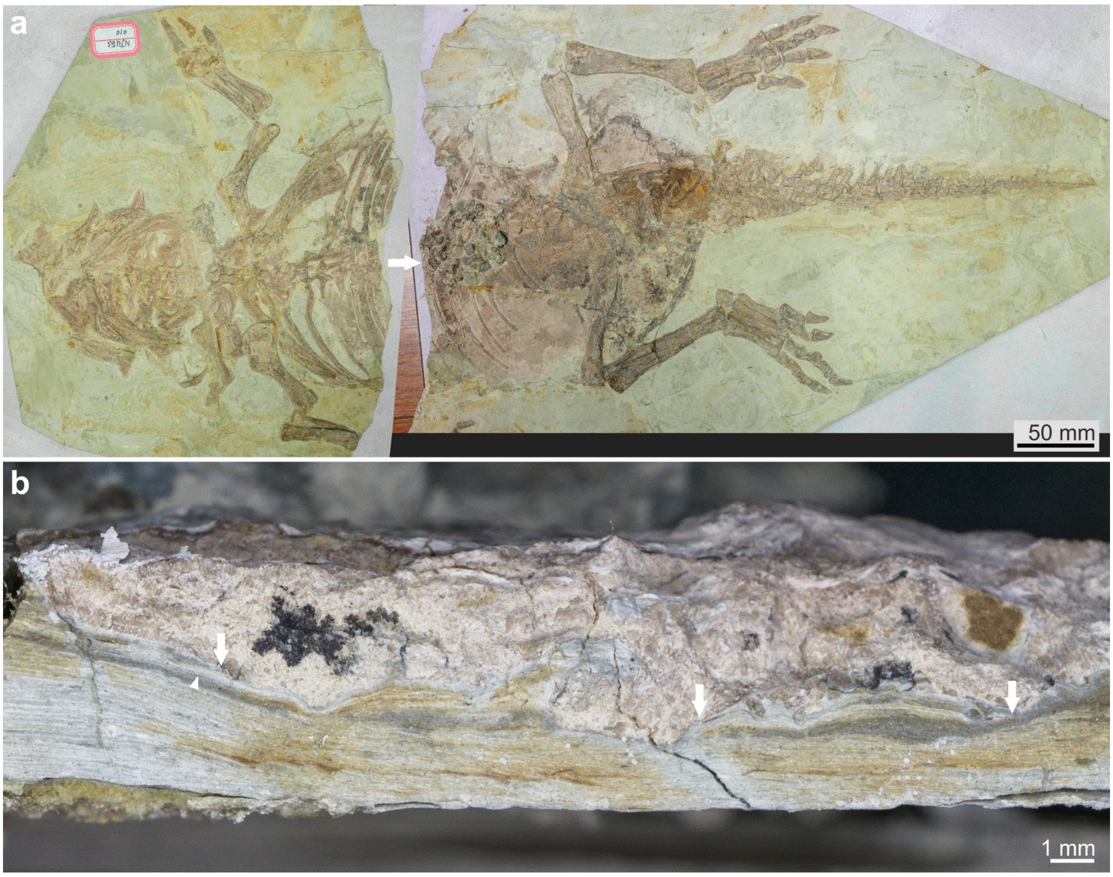

Supplementary Figure 1. Sedimentary structures associated with NJUES-10. a Overview of the specimen. Arrow indicates location of **e**. **b** Vertical section showing soft-sediment deformation of the laminae underlying the vertebrae. Arrows indicate lateral thinning and truncation of the laminae caused by the impacting carcass at deposition; arrowhead indicates normal grading in a sedimentary lamina.

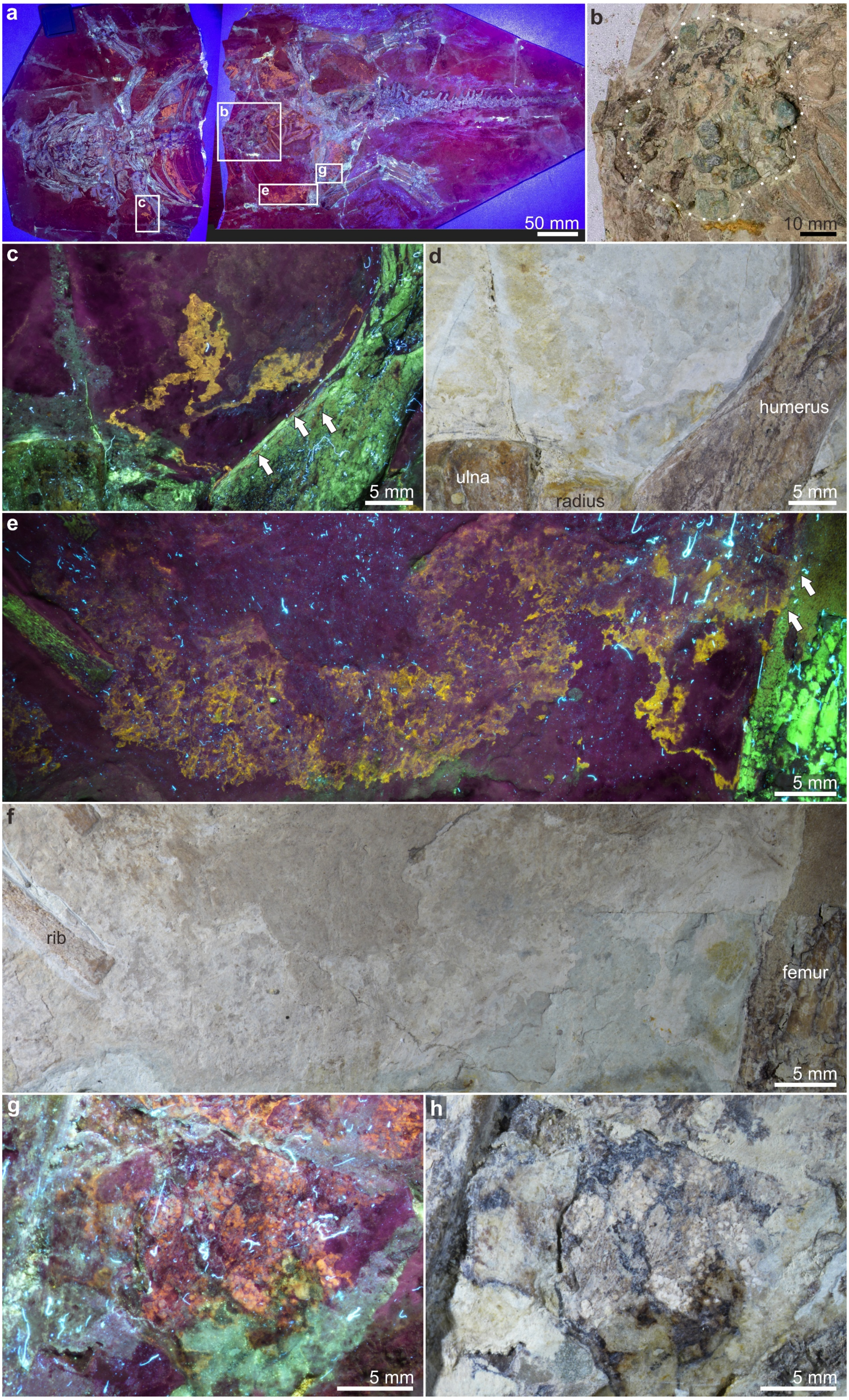
a
b
c
e
g
50 mm
10 mm
c
5 mm
d
ulna
radius
humerus
5 mm
e
5 mm
f
rib
femur
5 mm
g
5 mm
h
5 mm

Supplementary Figure 2. Preserved soft tissues of *Psittacosaurus* (NJUES-10). a Overview of the specimen in UV light showing distinct fluorescence hues for bone (green), soft tissues (yellow) and glue (bright cyan) against a dark purple sedimentary matrix. **b** Detail of the cluster of gastroliths under daylight. **c**–**h** Details of the soft tissues in the regions indicated in **a** under UV light (**c**, **e**, **g**) and daylight (**d**, **f**, **h**). Arrows in **c** and **e** indicate the fossil skin layer overlying the humerus (**c**; most of the overlying fossil skin was lost during fossil preparation) and femur (**e**).

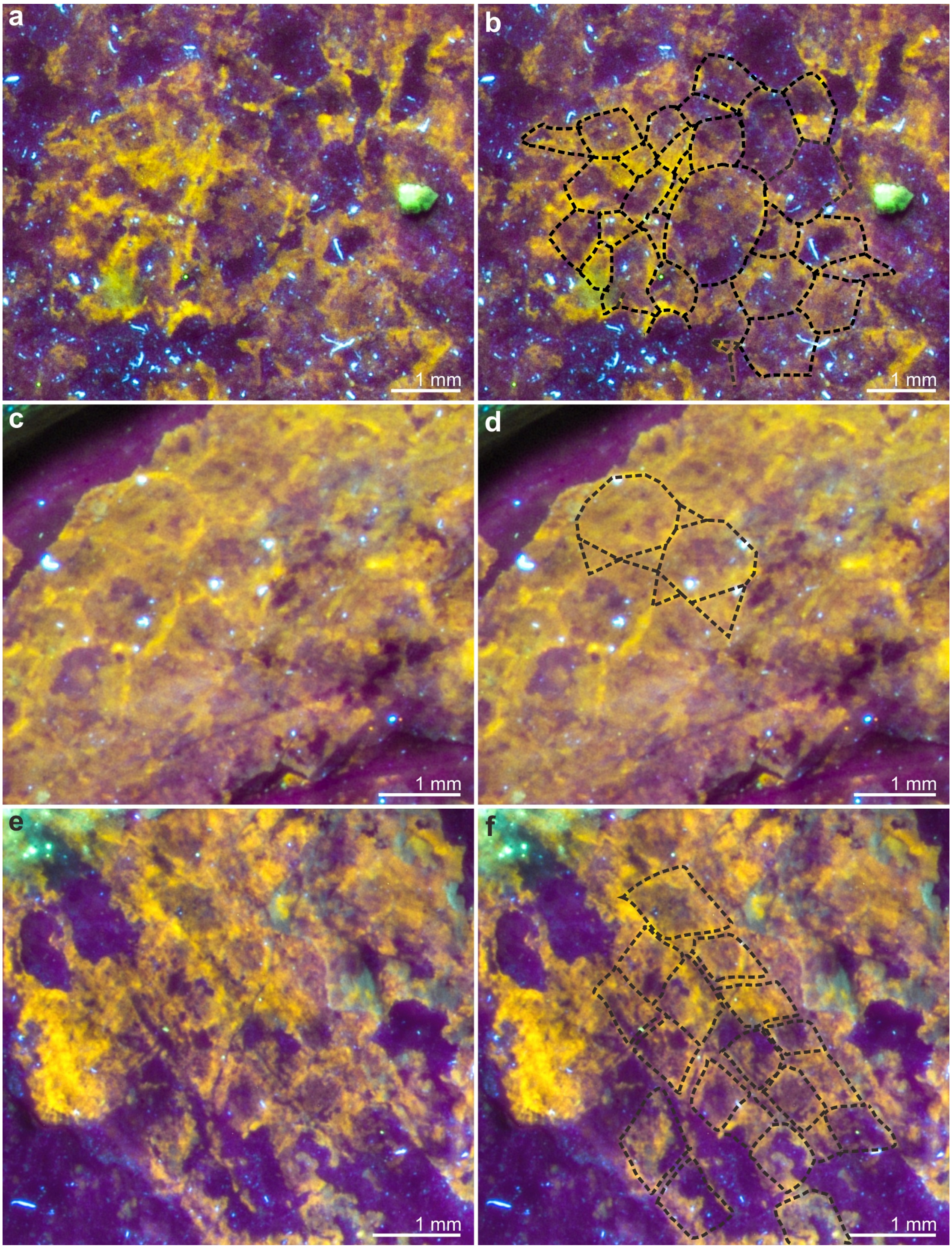

Supplementary Figure 3. UV images with interpretive drawings (dashed lines) showing variation in preserved scale morphology. a–b Central rounded feature scale surrounded by smaller, polygonal-to-rounded basement scales. **c–d** Hexagram pattern of basement scales, including a central polygonal scale and surrounding smaller triangular scales. **e–f** Quadrangular scales forming distinct rows.

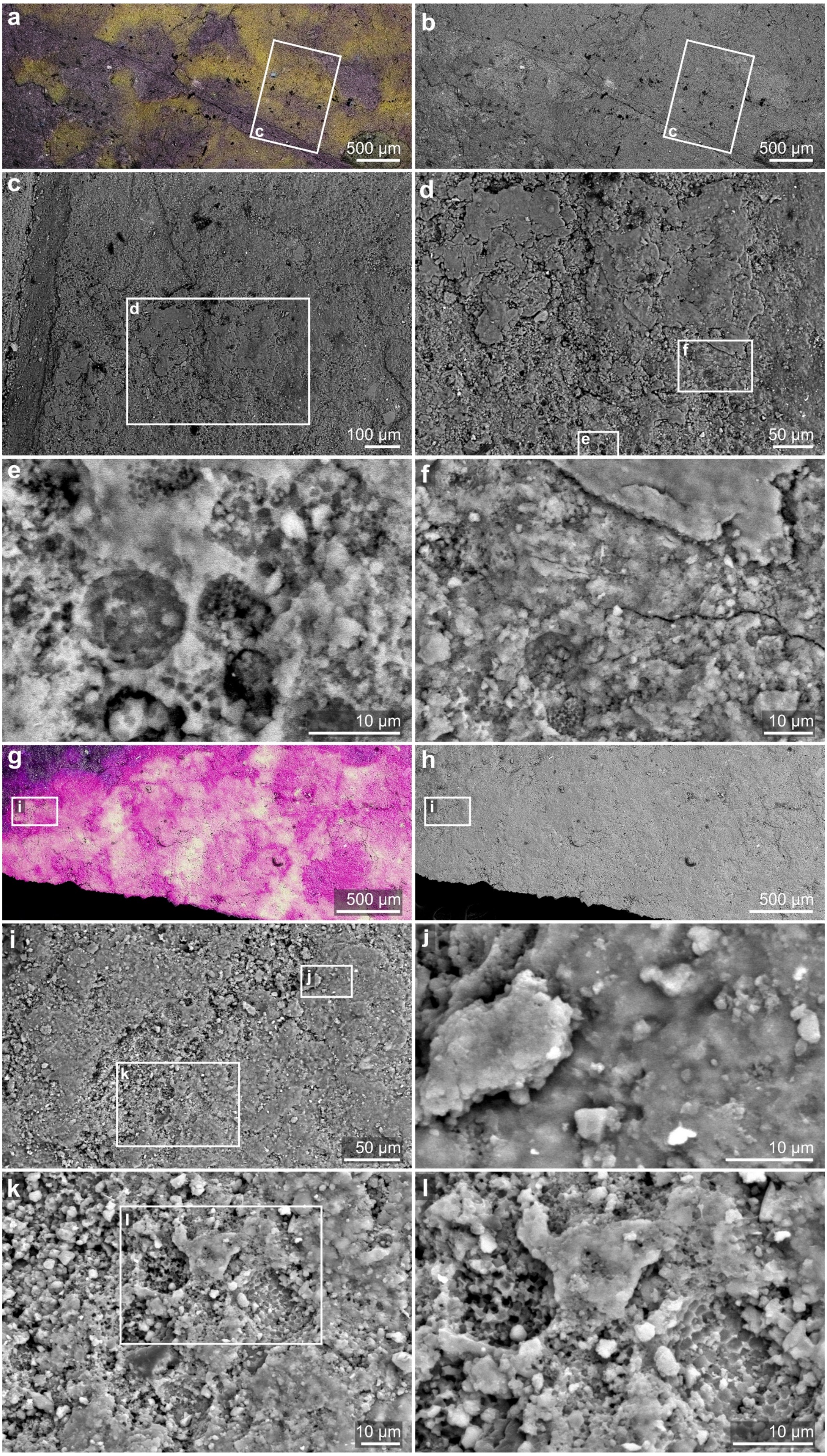
a
c
500 µm
b
c
500 µm
c
d
100 µm
d
f
e
50 µm
e
10 µm
f
10 µm
g
i
500 µm
h
i
500 µm
i
j
k
50 µm
j
10 µm
k
l
10 µm
l
10 µm

Supplementary Figure 4. Ultrastructure of the fossil skin surface. a–b Superimposed SEM and UV images (**a**) and SEM image of the same region (**b**). **c–d** Fossil skin showing multiple layers, with a slightly uneven surface. **e–f** Detailed views showing hemispherical depressions that comprise aggregates of cubic to subspherical voids of varying sizes. **g–h** Superimposed SEM and UV images (**g**) and SEM image of the same region (**h**). **i–l** Detailed views showing amorphous to fine-grained texture of the fossil skin surface (**j**) and hemispherical depressions that comprise aggregates of cubic to subspherical voids (**k–l**).

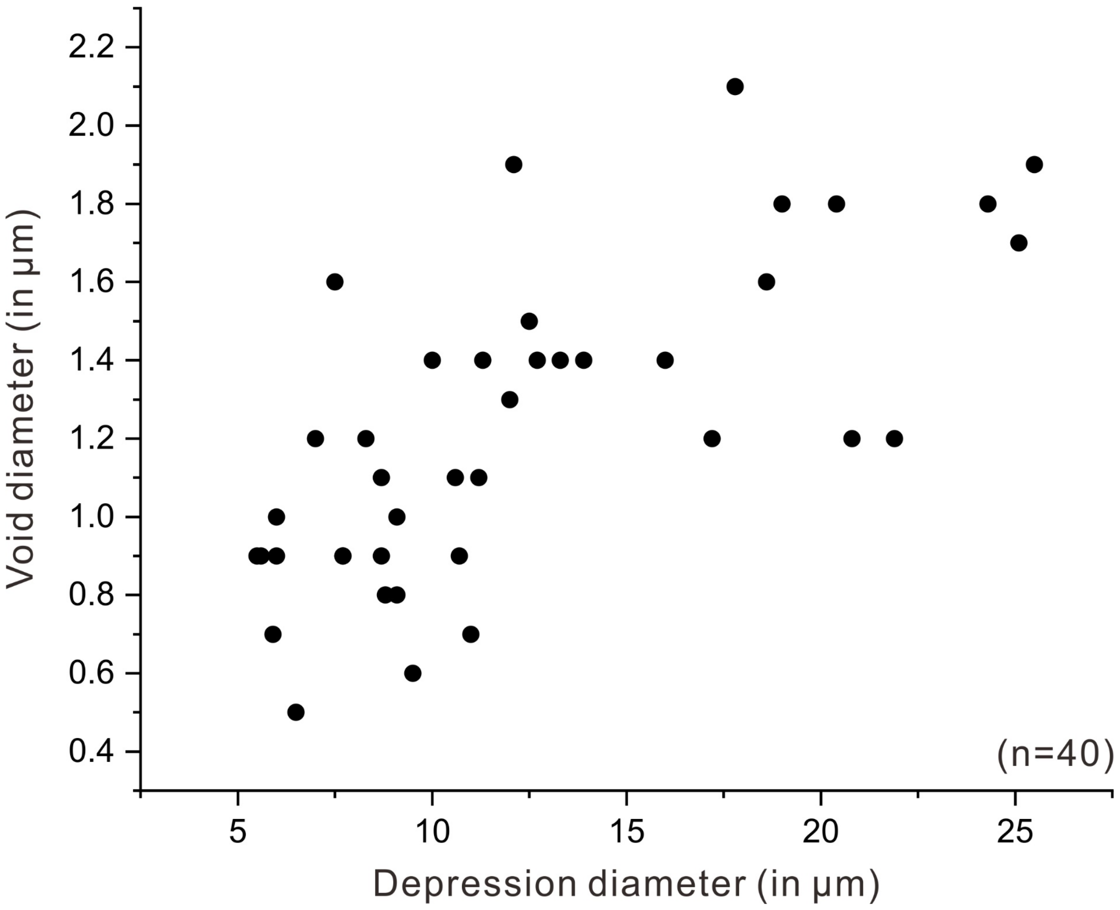

Supplementary Figure 5. Scatter plot of data on the diameter of the hemispherical depressions and of their constituent voids.

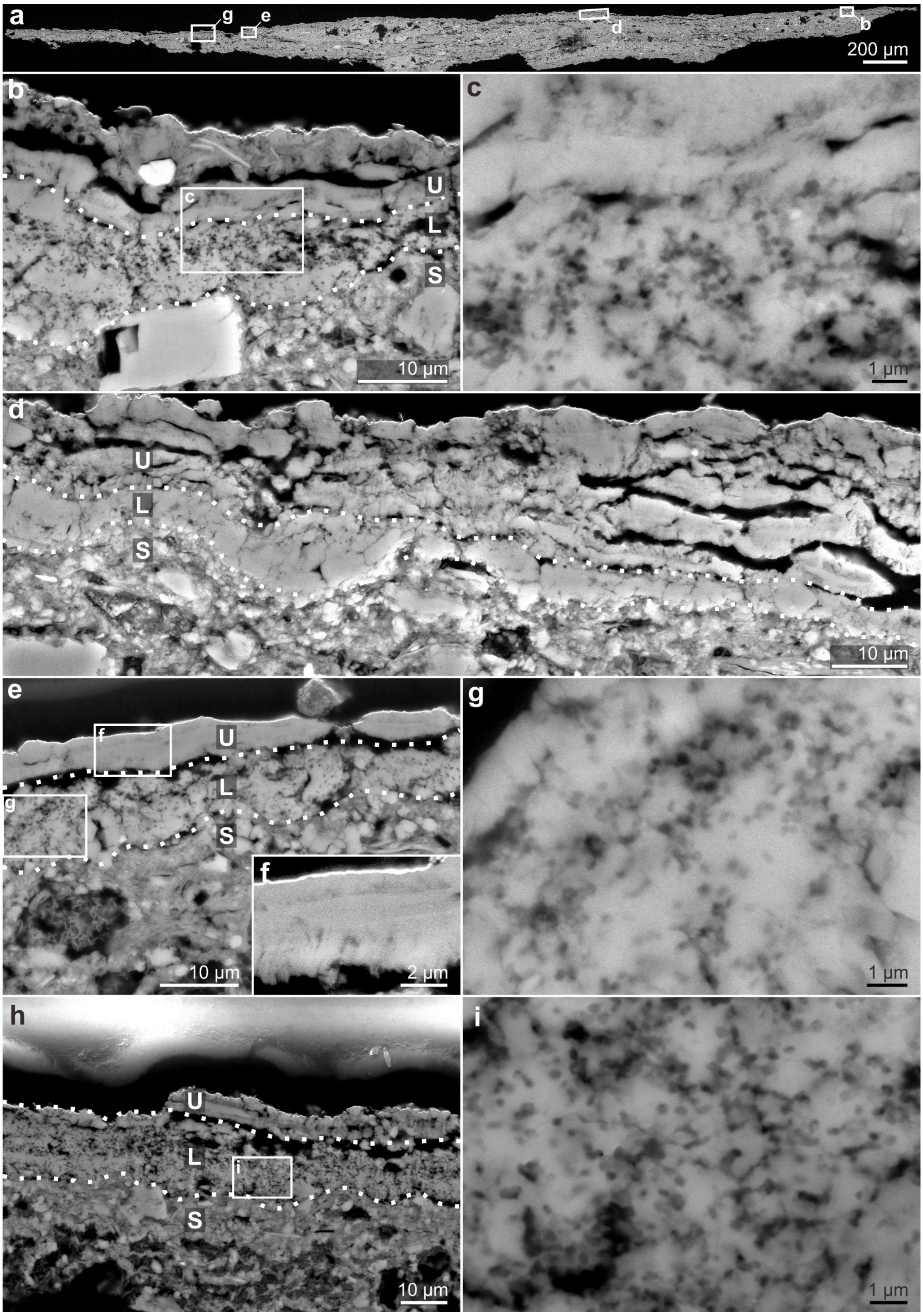

Supplementary Figure 6. Overview (a) and details (b–i) of a polished vertical section of the fossil skin showing densely packed mouldic melanosomes in the lower skin layer. Note that the upper skin layer is lined by isopachous cement (**e**–**f**). The dashed lines in **b**, **d**, **e** and **h** denote boundaries among the upper (U) and lower (L) skin layers and the underlying sediment (S).

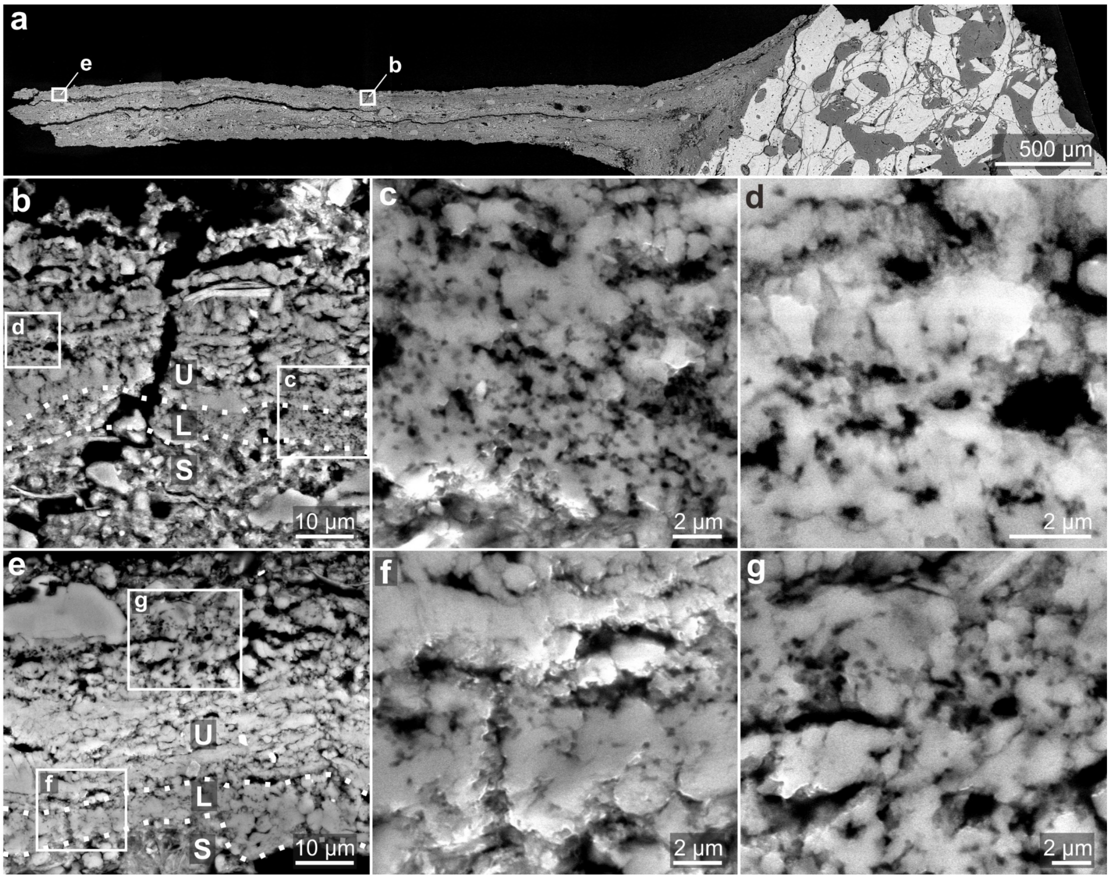

Supplementary Figure 7. Overview (a) and details (b–g) of a polished vertical section through the fossil skin showing mouldic melanosomes in upper and lower skin layers. The dashed lines in **b** and **e** denote boundaries among the upper (U) and lower (L) skin layers and the underlying sediment (S).

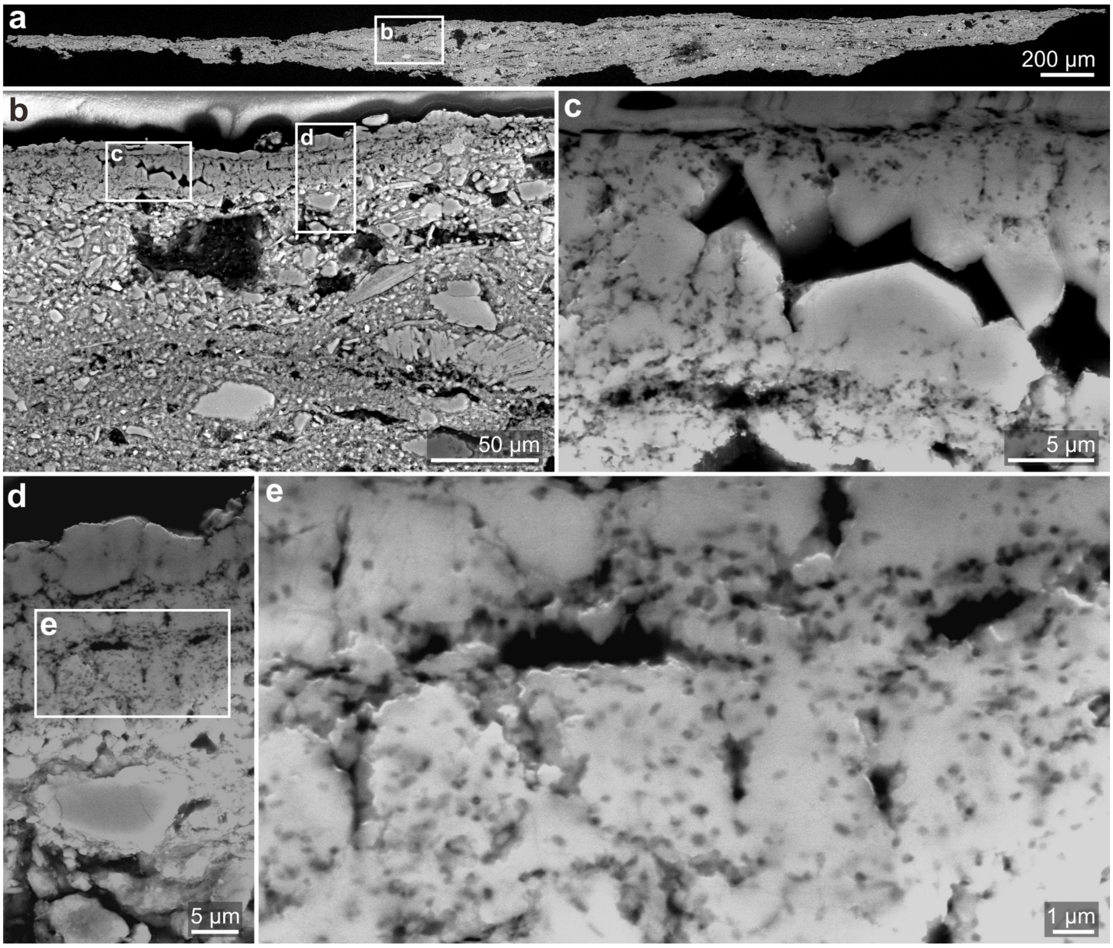

Supplementary Figure 8. Overview (a) and details (b–e) of a polished vertical section through the fossil skin showing mouldic melanosomes. Note absence of melanosomes in the large silica crystals in the lower skin layer (c).

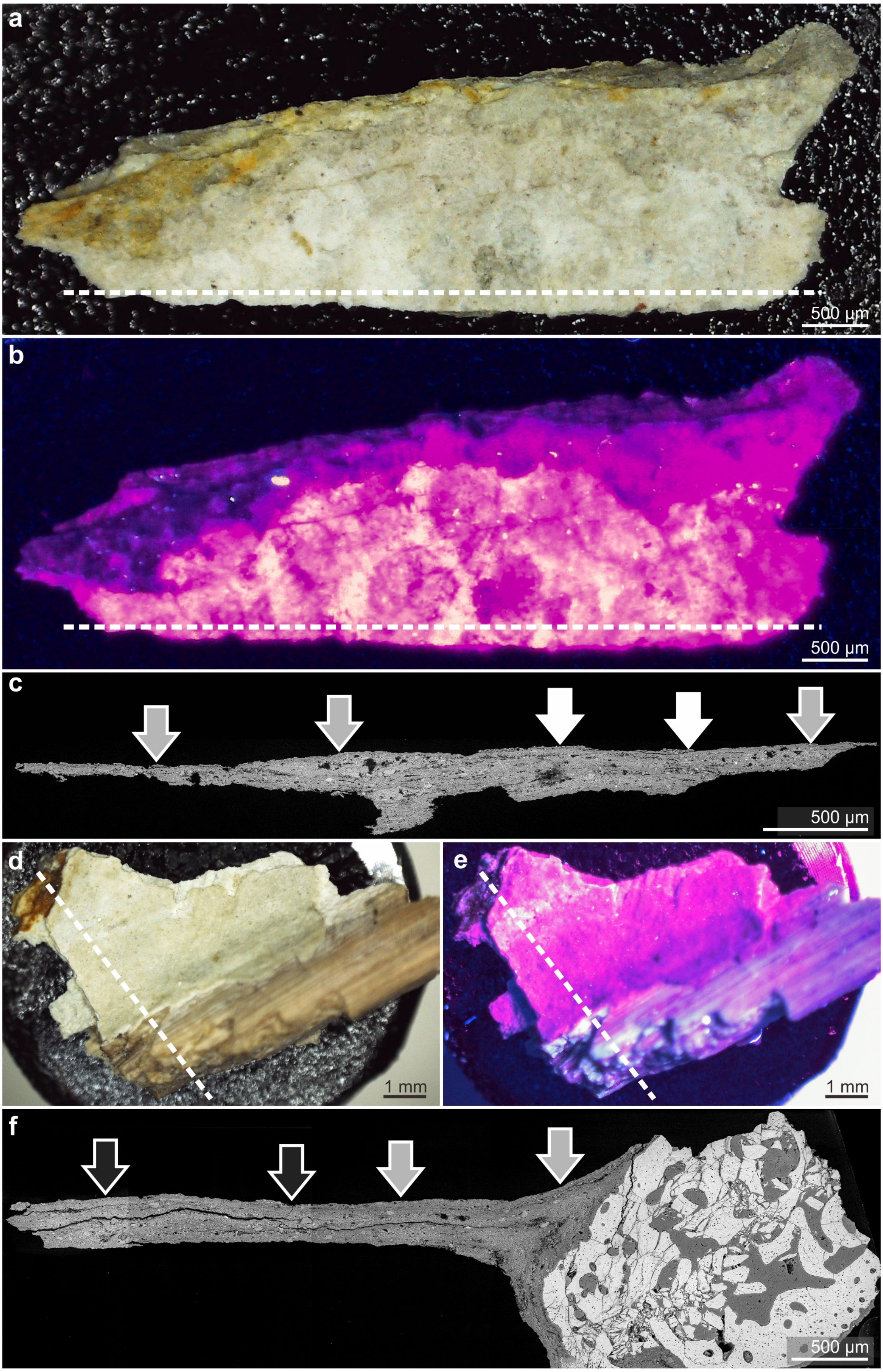

Supplementary Figure 9. Lateral variation of melanosome distribution in the fossil skin samples. a–b Fossil skin sample (sampling location shown in Fig. 2**e**)

under daylight (**a**) and UV (**b**) light; dashed lines indicate the approximate position of the polished section. **c** SEM image of the polished section; the white and grey arrows indicate absence of melanosomes and melanosomes present in only the lower skin layer, respectively. **d**–**e** A fossil skin sample (sampling location shown in Fig. 2**b**) under daylight (**d**) and UV (**e**) light; dashed lines indicate the approximate position of the polished section. **f** SEM image of the polished section; the black and grey arrows indicate melanosomes present in both the upper and lower skin layers and melanosomes present in only the lower skin layer, respectively.

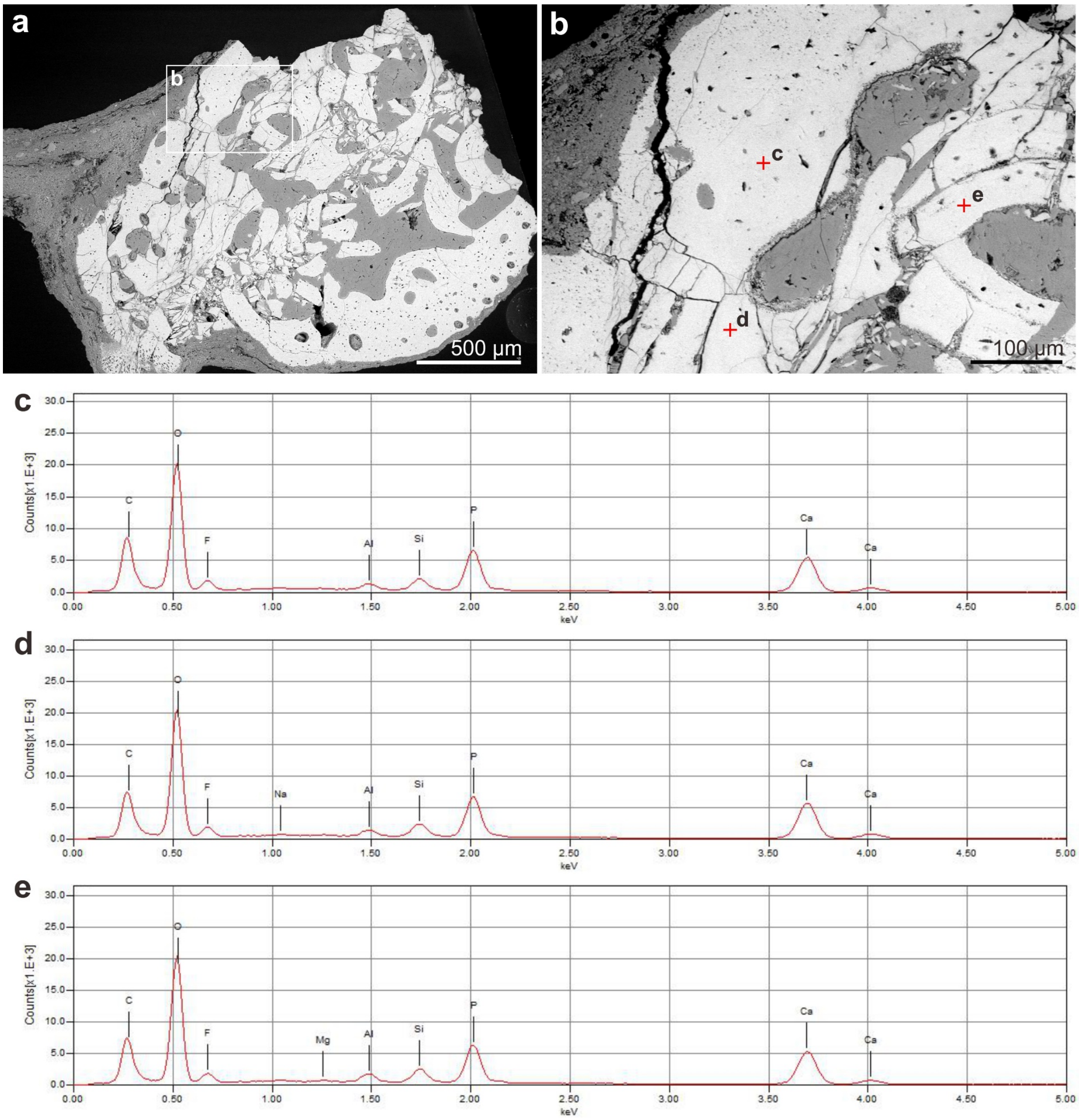

Supplementary Figure 10. Backscattered electron micrographs (a–b) and EDS X-ray spectra (c–e) of a *Psittacosaurus* rib.

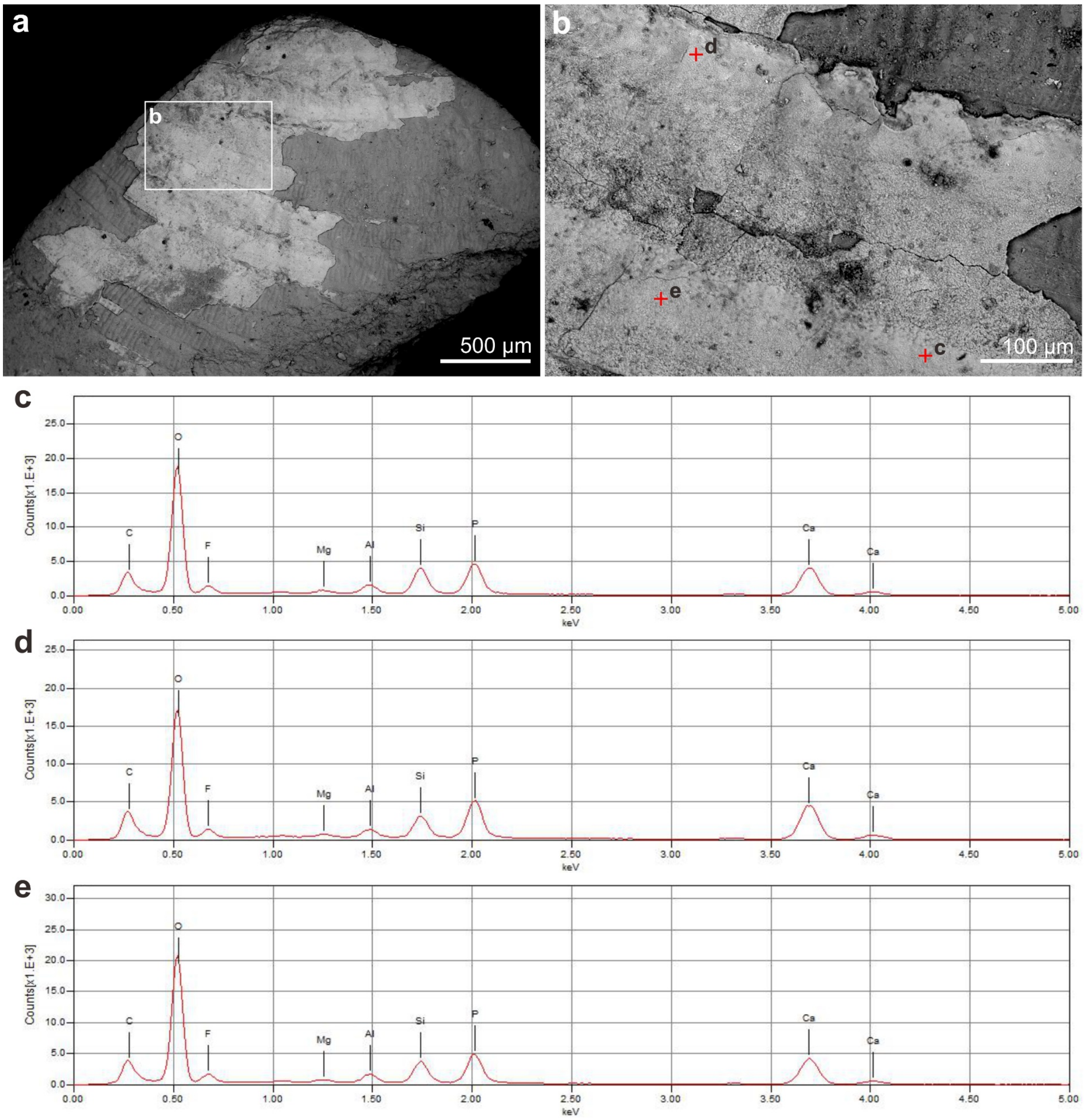

Supplementary Figure 11. Backscattered electron micrographs (a–b) and EDS X-ray spectra (c–e) of clam shrimp shell remains from the same bedding plane as the fossil skin.

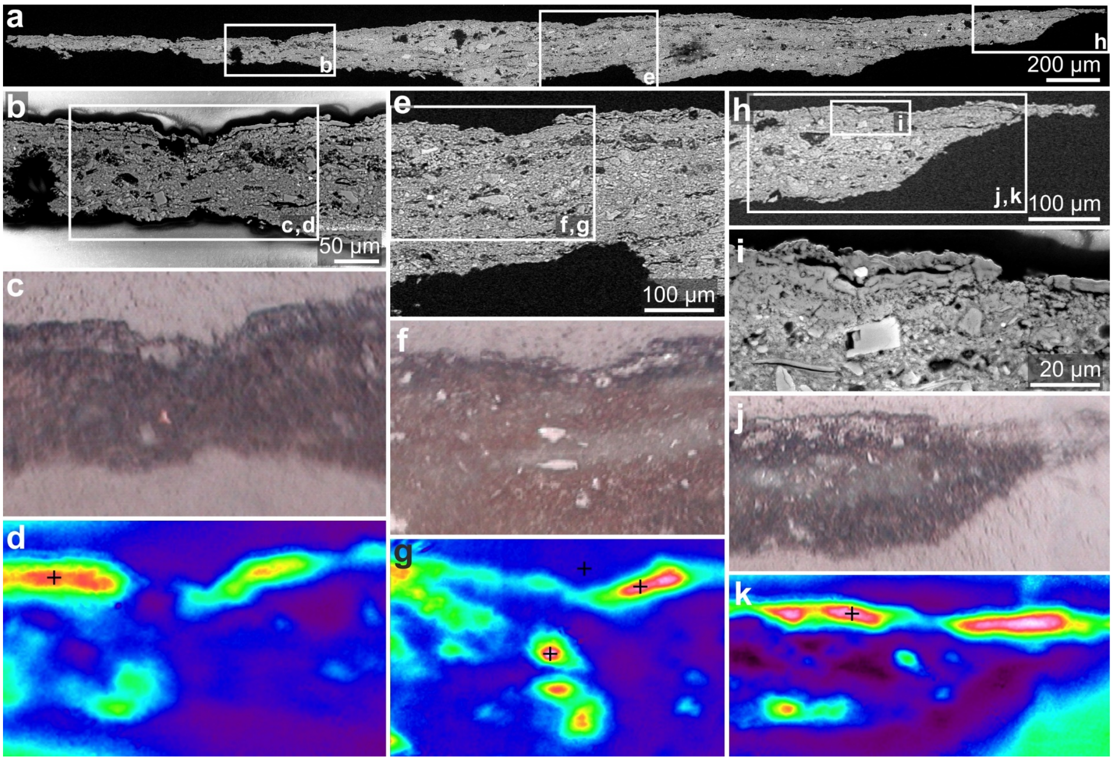

Supplementary Figure 12. µ-FTIR absorbance mapping of the fossil skin. a Backscattered electron micrograph showing an overview of a polished vertical section through the fossil skin. **b**, **e**, **h**, **i** Details of the fossil skin and the underlying sediment. **c**–**d**, **f**–**g** and **j**–**k**, Light micrographs and corresponding µ-FTIR maps of the three regions indicated in **a**. Warm colours in µ-FTIR maps indicate high absorbance; the crosses in µ-FTIR maps indicate locations of individual spectra shown in Supplementary Fig. 13.

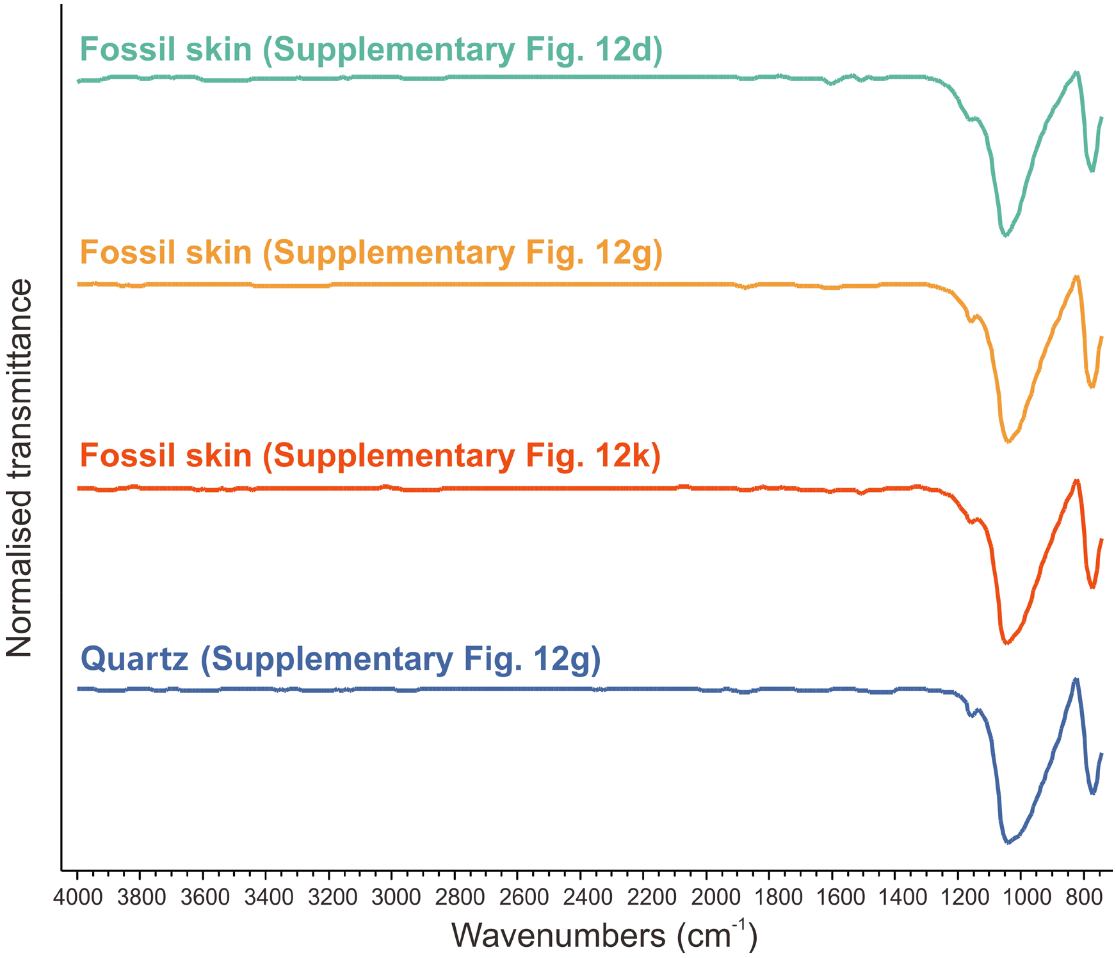

Supplementary Figure 13. Representative µ-FTIR transmittance spectra of the fossil skin and quartz grain from the regions shown in Supplementary Fig. 12. Spot locations of individual spectra are shown as crosses in Supplementary Fig. 12.

	Specimen number	Humerus	Radius	Ulna	Femur	Tibia	Fibula	FL	HL	FL/HL Ratio	Age
	NJUES-10	67	46	46	78	90	?	113	168	0.673	
Hatchling	IVPP V16902.1	22	18	18	22	25	24	40	47	0.851	<1
	IVPP V16902.2	24	20	20	25	27	25	44	52	0.846	<1
	IVPP V16902.3	25	21	21	26	29	30	46	55	0.836	<1
Juvenile	ELDM V1037	32	26	28	38	44	48	58	82	0.707	1
	ELDM V1038.21	38	30	32	44	48	53	68	92	0.739	2
	ELDM V1038.15	39	31	30	46	51	54	70	97	0.721	2
	ElDM V1038.11	40	31	31	47	52	56	71	99	0.717	2
	ElDM V1038	38	28	30	47	50	52	66	97	0.68	2
	IVPP V14341.4	50	32	35	62	65	70	82	127	0.646	2
	IVPP V14341.1	62	40	42	73	82	82	102	155	0.658	3
	IVPP V14342	64	41	44	81	88	89	105	169	0.621	?
Subadult	IVPP V14748	90	54	59	109	119	118	144	228	0.633	5
	IVPP V14749	90	56	65	117	125	122	146	242	0.604	5
	IVPP V18343	94	64	74	132	135	135	158	267	0.592	7
	IVPP V18344	108	75	75	145	150	149	183	295	0.62	7
Adult	IVPP V12716	137	85	94	162	175	165	222	337	0.659	10

Supplementary Table 1 | Measurements of NJUES-10 long bones and comparison with other *Psittacosaurus* specimens. Data for the other specimens were taken from ref. [4] in the Supplementary information. Abbreviations: 'FL', forelimb (humerus plus radius); 'HL', hindlimb (femur plus tibia). All measurements are lengths in mm.

Supplementary References

1. Sereno, P.C. Psittacosauridae. In: Weishampel, D.B., Dodson, P., Osmolska, H. (eds.) The Dinosauria, pp. 579–592. University of California Press, 1990.
2. Sereno, P. C. Taxonomy, cranial morphology, and relationships of parrot-beaked dinosaurs (Ceratopsia: *Psittacosaurus*). In: Ryan, M.J., Chinnery-Allgeier B.J., Eberth, D.A. (eds) New Perspectives on Horned Dinosaurs, pp. 21–58. Indiana University Press, 2010.
3. Hedrick, B. P. & Dodson, P. Lujiatun Psittacosaurids: understanding individual and taphonomic variation using 3D geometric morphometrics. *PLoS ONE* **8**, e69265 (2013).
4. Zhao, Q., Benton, M.J., Sullivan, C., Sander, M.P. & Xu, X. Histology and postural change during the growth of the ceratopsian dinosaur *Psittacosaurus lujiatunensis*. *Nat. Commun.* **4**, 1–8 (2013).
5. Erickson, G.M., Makovicky, P.J., Inouye, B.D., Zhou, C.F. & Gao, K. Q. A life table for *Psittacosaurus lujiatunensis*: initial insights into ornithischian dinosaur population biology. *Anat. Rec.* **292**, 1684–1684 (2009).
6. Hendrickx, C. et al. Morphology and distribution of scales, dermal ossifications, and other non-feather integumentary structures in non-avialan theropod dinosaurs. *Biol. Rev.* **97**, 960–1004 (2022).
7. Bell, P.R., Hendrickx, C., Pittman, M., Kaye, T.G. & Mayr, G. The exquisitely preserved integument of *Psittacosaurus* and the scaly skin of ceratopsian dinosaurs. *Commun. Biol.* **5**, 809 (2022).
8. Vinther, J. et al. 3D camouflage in an ornithischian dinosaur. *Curr. Biol.* **26**, 2456–2462 (2016).
9. Alibardi, L. Histology, ultrastructure, and pigmentation in the horny scales of growing crocodilians. *Acta Zool.* **92**, 187–200 (2011).
10. Li, Q. et al. Melanosome evolution indicates a key physiological shift within feathered dinosaurs. *Nature* **507**, 350–353 (2014).
11. Rossi, V., McNamara, M.E., Webb, S.M., Ito, S. & Wakamatsu, K. Tissue-specific geometry and chemistry of modern and fossilized melanosomes reveal internal anatomy of extinct vertebrates. *Proc. Natl. Acad. Sci. USA* **116**, 17880–17889 (2019).
12. Pinheiro, F.L. et. al. Chemical characterization of pterosaur melanin challenges color inferences in extinct animals. *Sci. Rep.* **9**, 15947 (2019).
13. Mayr, G., Peters, S.D., Plodowski, G. & Vogel, O. Bristle-like integumentary structures at the tail of the horned dinosaur *Psittacosaurus*. *Naturwissenschaften* **89**, 361–365 (2002).
14. Slagter, S., Tarhan, L.G., Hao, W., Planavsky, N.J. & Konhauser, K.O. Experimental evidence supports early silica cementation of the Ediacara Biota. *Geology* **49**, 51–55 (2021).
15. Lange, L., Huang, Y., & Busk, P. K. Microbial decomposition of keratin in nature—a new hypothesis of industrial relevance. *Appl. Microbiol. Biotechnol.* **100**, 2083–2096 (2016).
16. Butler, A.D., Cunningham, J.A., Budd, G.E. & Donoghue, P.C. Experimental taphonomy of *Artemia* reveals the role of endogenous microbes in mediating decay and fossilization. *Proc. R. Soc. B: Biol. Sci.* **282**, p.20150476 (2015).
