# Supplementary material for: Cellular structure of dinosaur scales reveals retention of reptile-type skin during the evolutionary transition to feathers
Source: Nat Commun. 2024 May 21;15:4063. doi: 10.1038/s41467-024-48400-3 (PMC11109146; doi:10.1038/s41467-024-48400-3)
Supplement: Supplementary file 3 — Reporting Summary [file 41467_2024_48400_MOESM3_ESM.pdf]

nature portfolio

Corresponding author(s): Zixiao Yang

Last updated by author(s): Apr 16, 2024

Reporting Summary

Nature Portfolio wishes to improve the reproducibility of the work that we publish. This form provides structure for consistency and transparency in reporting. For further information on Nature Portfolio policies, see our Editorial Policies and the Editorial Policy Checklist.

Statistics

For all statistical analyses, confirm that the following items are present in the figure legend, table legend, main text, or Methods section.

n/a	Confirmed	
☐	☒	The exact sample size (*n*) for each experimental group/condition, given as a discrete number and unit of measurement
☐	☒	A statement on whether measurements were taken from distinct samples or whether the same sample was measured repeatedly
☒	☐	The statistical test(s) used AND whether they are one- or two-sided<br>*Only common tests should be described solely by name; describe more complex techniques in the Methods section.*
☒	☐	A description of all covariates tested
☒	☐	A description of any assumptions or corrections, such as tests of normality and adjustment for multiple comparisons
☒	☐	A full description of the statistical parameters including central tendency (e.g. means) or other basic estimates (e.g. regression coefficient) AND variation (e.g. standard deviation) or associated estimates of uncertainty (e.g. confidence intervals)
☒	☐	For null hypothesis testing, the test statistic (e.g. *F*, *t*, *r*) with confidence intervals, effect sizes, degrees of freedom and *P* value noted<br>*Give P values as exact values whenever suitable.*
☒	☐	For Bayesian analysis, information on the choice of priors and Markov chain Monte Carlo settings
☒	☐	For hierarchical and complex designs, identification of the appropriate level for tests and full reporting of outcomes
☒	☐	Estimates of effect sizes (e.g. Cohen's *d*, Pearson's *r*), indicating how they were calculated

Our web collection on statistics for biologists contains articles on many of the points above.

Software and code

Policy information about availability of computer code

Data collection	SpectrumIMAGE R1.11.2.0016 (https://www.perkinelmer.com/product/spotlight-400-s-w-kit-sp400-lx108895)
Data analysis	SpectrumIMAGE R1.11.2.0016 (https://www.perkinelmer.com/product/spotlight-400-s-w-kit-sp400-lx108895) and SpectraGryph v1.2.16.1 (https://www.effemm2.de/spectragryph/)

For manuscripts utilizing custom algorithms or software that are central to the research but not yet described in published literature, software must be made available to editors and reviewers. We strongly encourage code deposition in a community repository (e.g. GitHub). See the Nature Portfolio guidelines for submitting code & software for further information.

Data

Policy information about availability of data

All manuscripts must include a data availability statement. This statement should provide the following information, where applicable:

- Accession codes, unique identifiers, or web links for publicly available datasets
- A description of any restrictions on data availability
- For clinical datasets or third party data, please ensure that the statement adheres to our policy

The data supporting the findings of this study are available within the paper and the Supplementary Information. Source data are provided with this paper.

Research involving human participants, their data, or biological material

Policy information about studies with human participants or human data. See also policy information about sex, gender (identity/presentation), and sexual orientation and race, ethnicity and racism.

Reporting on sex and gender	Not applicable as our research does not involve human participants or human data.
Reporting on race, ethnicity, or other socially relevant groupings	Not applicable as our research does not involve human participants or human data.
Population characteristics	Not applicable as our research does not involve human participants or human data.
Recruitment	Not applicable as our research does not involve human participants or human data.
Ethics oversight	Not applicable as our research does not involve human participants or human data.

Note that full information on the approval of the study protocol must also be provided in the manuscript.

Field-specific reporting

Please select the one below that is the best fit for your research. If you are not sure, read the appropriate sections before making your selection.

☐ Life sciences ☐ Behavioural & social sciences ☒ Ecological, evolutionary & environmental sciences

For a reference copy of the document with all sections, see nature.com/documents/nr-reporting-summary-flat.pdf

Ecological, evolutionary & environmental sciences study design

All studies must disclose on these points even when the disclosure is negative.

Study description	We systematically examined the preserved skin of a feathered dinosaur, including the gross morphology, ultrastructure, elemental composition and FTIR signature of the preserved skin.
Research sample	Among feathered dinosaurs, Psittacosaurus is an early-diverging dinosaur with both scales and primitive feathers. Understanding its integumentary anatomy therefore is critical to understanding the evolutionary transition from scales to feathers. The specimen NJUES-10 belongs to the Early Cretaceous Jehol Biota of China and likely represents a juvenile individual. The specimen is preserved on a single, fragmented slab that has been glued together along the fissures. The specimen was examined for evidence of preserved skin using a Nikon SMZ25 stereomicroscope coupled with a UV light source (wavelength 365 nm). Under UV light, discontinuous patches of soft tissue can be readily distinguished from the bones, sediment and glue via distinct differences in fluorescence colour. Regions of soft tissue lacking obvious contamination by glue were selected for sampling.
Sampling strategy	No sample size calculation was performed. Small samples (2–5 mm wide) of preserved skin and the enclosing sediments were dissected from the specimen. All body parts that preserved skin remains were examined, including regions of the limbs, chest and abdomen. Sampling of additional specimens was not feasible due to limited access and temporal and financial constraints.
Data collection	Scanning electron microscopy (SEM): SEM analyses used a JEOL IT-100 variable pressure (VP)-SEM equipped with a backscatter detector and a 30 mm2 EDS detector. Samples were examined at accelerating voltages of 10–20 kV and a working distance of 10 mm. Most samples were uncoated for SEM analyses in VP mode; selected samples were sputter-coated with Au for high-resolution imaging in high-vacuum mode.<br><br>Micro-attenuated total refection Fourier-transform infrared (µATR-FTIR) spectroscopy: Infrared transmittance spectra were collected from regions of interest in polished vertical sections of the fossil skin in the School of Biological, Earth and Environmental Sciences, University College Cork. Data collection used a Perkin Elmer Spotlight 400i FTIR microscope coupled to a Frontier spectrometer, a dedicated high-resolution ATR Ge imaging accessory and a computer. Collection was via the software SpectrumIMAGE R1.11.2.0016 and the parameters were set as follows: resolution 16 cm-1, 32 scans per pixel, interferometer speed 1.0 cm/s, scan region 4000 cm-1 to 750 cm-1 and pixel size 1.56 µm. The resulting transmittance maps contain an infrared spectrum for each pixel. A background spectrum was collected prior to each map to account for signal contribution from the instrument and environment. Raw spectra were processed as follows: atmospheric correction (to compensate for water vapor and CO2 contributions) in SpectrumIMAGE R1.11.2.0016 and baseline correction for extracted spectra in SpectraGryph v1.2.16.1 (using the default coarseness and offset values in the advanced baseline correction function).<br><br>SEM and µ-FTIR data were collected by Zixiao Yang and Maria McNamara.
Timing and spatial scale	Regions of soft tissue lacking obvious contamination by glue were selected and sampled in August 2021. SEM and µ-FTIR data were collected during October 2021–June 2023. The regions analysed for SEM and µ-FTIR are ultrastructural in order to reveal the anatomy of the preserved skin. The data were not collected with a time scale.
Data exclusions	No data were excluded from the analyses.

Reproducibility	Image acquisition of the scanning electron micrographs (Figs. 3d–h, 4c–g, 5c–e, g–p, and the first panel of 6 and Supplementary Figs. 4, 6–8, 9c, f, 10a–b, 11a–b and 12a–b, e, h–i) followed convention in the field. These micrographs were obtained as single, unique, images. Repeated acquisition of images in the same region is not standard procedure as it may lead to beam damage.
Randomization	Not relevant as this is not an experimental study.
Blinding	Not relevant as this is not an experimental study.

Did the study involve field work? ☐ Yes ☒ No

Reporting for specific materials, systems and methods

We require information from authors about some types of materials, experimental systems and methods used in many studies. Here, indicate whether each material, system or method listed is relevant to your study. If you are not sure if a list item applies to your research, read the appropriate section before selecting a response.

Materials & experimental systems

n/a	Involved in the study
☒	☐ Antibodies
☒	☐ Eukaryotic cell lines
☐	☒ Palaeontology and archaeology
☒	☐ Animals and other organisms
☒	☐ Clinical data
☒	☐ Dual use research of concern
☒	☐ Plants

Methods

n/a	Involved in the study
☒	☐ ChIP-seq
☒	☐ Flow cytometry
☒	☐ MRI-based neuroimaging

Palaeontology and Archaeology

Specimen provenance	The studied Psittacosaurus specimen NJUES-10 belongs to the fossil collections of the School of Earth Sciences and Engineering, Nanjing University, Nanjing, China. Permission for studying and sampling of the specimen was granted by the school in 2021.
Specimen deposition	The specimen is hosted in the School of Earth Sciences and Engineering, Nanjing University, which is a public, accessible and recognised repository.
Dating methods	No new dates are provided.

☐ Tick this box to confirm that the raw and calibrated dates are available in the paper or in Supplementary Information.

Ethics oversight	No ethical approval or guidance was required as the specimen was not recovered in the field.

Note that full information on the approval of the study protocol must also be provided in the manuscript.

Plants

Seed stocks	*Report on the source of all seed stocks or other plant material used. If applicable, state the seed stock centre and catalogue number. If plant specimens were collected from the field, describe the collection location, date and sampling procedures.*
Novel plant genotypes	*Describe the methods by which all novel plant genotypes were produced. This includes those generated by transgenic approaches, gene editing, chemical/radiation-based mutagenesis and hybridization. For transgenic lines, describe the transformation method, the number of independent lines analyzed and the generation upon which experiments were performed. For gene-edited lines, describe the editor used, the endogenous sequence targeted for editing, the targeting guide RNA sequence (if applicable) and how the editor was applied.*
Authentication	*Describe any authentication procedures for each seed stock used or novel genotype generated. Describe any experiments used to assess the effect of a mutation and, where applicable, how potential secondary effects (e.g. second site T-DNA insertions, mosiacism, off-target gene editing) were examined.*
